# Cystic Fibrosis: Understanding Cystic Fibrosis Transmembrane Regulator Mutation Classification and Modulator Therapies

**DOI:** 10.3390/arm92040026

**Published:** 2024-07-20

**Authors:** Saba Anwar, Jin-Liang Peng, Kashif Rafiq Zahid, Yu-Ming Zhou, Qurban Ali, Chong-Rong Qiu

**Affiliations:** 1 Centre for Applied Molecular Biology, University of the Punjab Lahore, Lahore 53700, Pakistan; sabagemini5@gmail.com; 2 Department of Emergency, The Affiliated Ganzhou Hospital of Nanchang University, Ganzhou 341000, China; fengruyi_2004@163.com (J.-L.P.); 18007079761@163.com (Y.-M.Z.); 3 Department of Radiation Oncology, Melvin and Bren Simon Comprehensive Cancer Center, Indiana University School of Medicine, Indianaapolis, IN 46202, USA; kashifrafiq1989@gmail.com; 4 Department of Plant Breeding and Genetics, Faculty of Agricultural Sciences, University of the Punjab, Lahore 54590, Pakistan

**Keywords:** cystic fibrosis, fibrosis transmembrane regulator, targeted mutation, pathophysiology, modulators

## Abstract

**Highlights:**

**What are the main findings?**

- The study identifies and categorizes the six main classes of CFTR mutations based on their functional effects.
- The study explores the emerging field of CFTR modulators, which are designed to restore CFTR function or mitigate its consequences. These modulators are characterized by their mode of action and the specific mutation class they target.

**What is the implication of the main finding?**

- By understanding the broad range of CFTR mutations and their impacts on disease pathophysiology, it is possible to establish tailored treatment strategies for CF patients.
- The study highlights the potential for precision medicine methods in CF therapy.

**Abstract:**

A common life-threatening hereditary disease, Cystic Fibrosis (CF), affects primarily Caucasian infants. High sweat-salt levels are observed as a result of a single autosomal mutation in chromosome 7 that affects the critical function of the cystic fibrosis transmembrane regulator (CFTR). For establishing tailored treatment strategies, it is important to understand the broad range of CFTR mutations and their impacts on disease pathophysiology. This study thoroughly investigates the six main classes of classification of CFTR mutations based on their functional effects. Each class is distinguished by distinct molecular flaws, such as poor protein synthesis, misfolding, gating defects, conduction defects, and decreased CFTR expression at the apical membrane. Furthermore, this paper focuses on the emerging field of CFTR modulators, which intend to restore CFTR function or mitigate its consequences. These modulators, which are characterized by the mode of action and targeted mutation class, have the potential to provide personalized therapy regimens in CF patients. This review provides valuable insights into the genetic basis of CF pathology, and highlights the potential for precision medicine methods in CF therapy by thoroughly investigating CFTR mutation classification and related modulators.

## 1. Introduction

Cystic fibrosis (CF), previously known as the “salty sweat disease”, continues to pose a significant hazard to health. This autosomal recessive condition, which primarily affects people of Caucasian heritage, presents serious life-threatening complications [1]. Mutations in the gene that codes for the cystic fibrosis transmembrane conductance regulator (CFTR) lead to this disease, but not all mutations in CFTR protein show the phenotypic effect of CF [2]. The CFTR channel is a cAMP-dependent chloride channel, and belongs to the ABC transporter protein family. The CFTR protein is primarily localized in the plasma membrane of epithelial cells, particularly in the apical membranes which line the upper and lower airways, sinuses, exocrine pancreas ducts, parts of the gastrointestinal tract, and vas deferens. The mutations occur on chromosome number 7 and lead to molecular defects in the channel, thereby causing improper and inefficient Cl^−^ transport across the apical membranes of the epithelia. In most of the cases, depending upon the type of mutation, the channel is not present in membranes of epithelia. The channel becomes impermeable or exhibits limited permeability to Cl^−^ and causes disruptions in the fluid and electrolyte balance. This imbalance causes alterations in mucus composition, and eventually leads to organ dysfunction and eventual death [3]. Although the most common clinical manifestation of CF is progressive lung disease, its clinical manifestations are quite widespread, including bronchiectasis, respiratory airway infections, pancreatic insufficiency, salty sweat, reproductive complications, and obstruction of the intestines [4,5,6]. CF occurs in mild, moderate, and severe forms, depending on the type of mutation. Several diagnostic tests have been developed to diagnose the disease and its intensity, the earliest being the sweat chloride test [7]. Soon after its discovery, CF was thought to be incurable. Still, with time, great advancements have been made in the therapeutics and treatment of CF [8].

## 2. Structure and Function of CFTR

**Figure 1:**
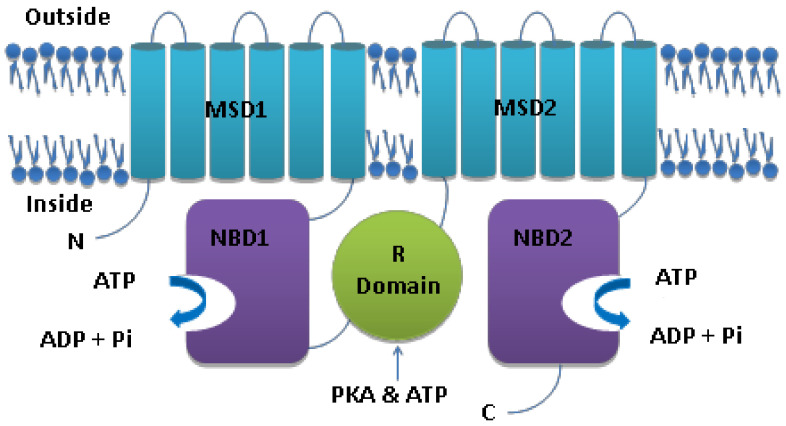
Structure of CFTR.

The amino acid sequence of the CFTR protein shows that it is a part of the ATP-binding cassette (ABC) Transporter protein family, which aids the transport of substrates against their concentration gradients. However, unlike the other ABC transporters, CFTR transports anions down their concentration gradients [9]. The CFTR protein functions are regulated using cAMP (Cyclic Adenosine Monophosphate) and transport anions (Figure 1), mainly chloride and bicarbonate, across the apical membranes of epithelial cells [10]. The predicted protein structure suggests that CFTR is a single polypeptide containing two transmembrane domains (TMD1 and TMD2) or membrane-spanning domains (MSD1 and MSD2), each of which comprises six hydrophobic transmembrane helices, TM1-6 in TMD1 and TM7-12 in TMD2 two nucleotide-binding domains (NBD1 and NBD2). Another unique feature of the CFTR, in contrast to other ABC proteins, is the presence of a cytoplasmic regulatory domain (R domain) in NBD1 and TMD2. The NBDs interact with ATP, and the R domain interacts with NBD1, separating the two TMDs [11,12]. The CFTR protein is expressed in the apical membranes of polarized epithelial cells in the sweat ducts, pancreatic duct, intestines, biliary tree, and vas deferens, and a few non-epithelial containing tissues such as erythrocytes, cardiac myocytes, immune cells such as macrophages, and smooth muscle [13,14,15,16,17,18]. The domains of the CFTR protein are aligned and arranged in a way to form a pore that selectively allows for the movement of anions [16]. In normal conditions, the CFTR gene is transcribed and translated to the CFTR membrane protein that functions exclusively as a chloride channel [19]. CFTR works as a transepithelial anion channel and transports chloride and bicarbonate ions [20,21,22].

Although CFTR is essentially known to function as an apical epithelial chloride channel, it indirectly influences the pH of proteins and co-regulates other ion transporters, most remarkably via the epithelial sodium channel (ENaC) present in the respiratory tracts and others, including calcium-activated chloride channels (CaCC), outward rectifying chloride channels (ORCC), the renal outer medullar K+ (ROMK) channels, and the sodium/proton exchanger NHE3, and an aquaporin channel [23,24]. Additionally, CFTR also accounts for the regulation of cellular processes such as endocytosis/exocytosis, ATP export, expression of pro-inflammatory immune cells such as cytokines, and pH regulations in the cells and vesicles [25,26]. 

## 3. Mechanism of Action of CFTR

It is widely believed that conformational changes in the channel occur as a result of ATP binding and hydrolysis. Phosphorylation of CFTR in cells is initiated by the activation of adenylate cyclase by a G-protein-associated hormone pathway, which triggers an increase in the cytoplasmic concentration of cAMP, activating PKA. Examples of pathways include acetylcholine, adenosine, epinephrine, glucagon, or beta-adrenergic agonists like isoproterenol and the vasoactive intestine peptide (VIP). It is believed that phosphorylation of the R domain enhances the channel activity and also stimulates the opening of CFTR channels. In contrast, the dephosphorylated R domain inhibits channel activity and promotes channel closure [16,27]. The CFTR channel is activated by phosphorylating the R domain at multiple sites by the enzyme Protein Kinase A (PKA). PKC can enhance the PKA-dependent activation of the channel by phosphorylating specific sites. Although intracellular cAMP is known to be the best modulator of the CFTR protein, in the intestine, certain cyclic-GMP dependent protein kinases are also known for activating and phosphorylation of CFTR. Unlike how most ion channels are affected by membrane potentials, CFTR is only slightly affected by it. The ATP concentration in the intracellular environment is maintained at approximately 2 mM, sufficient to ensure the activation of CFTR channels [3,11]. The universally accepted molecular model of CFTR gating suggests that the binding of ATP favors the dimerization of the NBDs (NBD1 and NBD2 interact with each other), which in turn causes changes in the TMDs/MSDs and leads to changes in the overall conformation of CFTR. Each Nucleotide-Binding Domain (NBD) contains a pair of nucleotide-binding sites capable of binding two ATP molecules. Upon ATP binding, the two NBDs converge, allowing the channel pore to open and chloride ions to pass through. The direction of ion flux is determined by the ion concentration gradient between intracellular and extracellular compartments. While one nucleotide-binding site has ATP hydrolysis activity, the other is dormant (degenerated site). Astonishingly, the energy liberated by ATP hydrolysis does not aid the chloride transport, and the CFTR protein channels can also be gated in the presence of ATP analogs, such as AMP-PNP, which are not even hydrolyzable. In all ABC proteins, the NBDs are known to bind and hydrolyze ATP, and there is significant evidence supporting the ATP-driven dimerization of NBDs [9,27,28].

## 4. CFTR Mutations and Cystic Fibrosis

**Figure 2:**
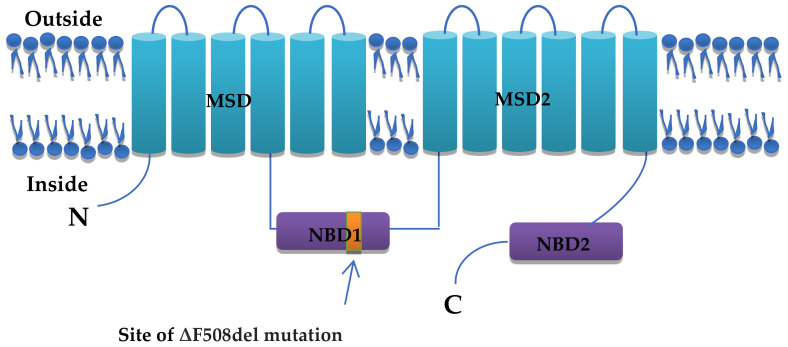
ΔF508 in CFTR.

Soon after discovering the CFTR gene, it was assumed that only a few disease-causing mutations would lead to the CF disease (Ratjen, 2009). However, around 2000 variants/mutations of the CFTR gene have been identified. Out of these variants, only 360 are disease-causing [29]. Nearly all of the CFTR mutations consist of a change in three or even less than three nucleotide base pairs that result in frame shifts, splice sites, nonsense mutations, or cause the substitutions of amino acids. Compared to the ΔF508, these mutations affected a much smaller percentage of cases, whereas ΔF508 is the first identified (Figure 2) as well as the most common present in approximately two-thirds (or 66%) of CF-causing alleles. “ΔF508” is a deletion of three base pairs, also referred to as F508del, which results in deleting a phenylalanine residue at amino acid position 508 [19,30].

## 5. Classification of CFTR Mutations

Several CF mutations, both disease-causing and non-disease-causing, have been identified. Typically, CF mutations are divided into six classes (I-VI) based on their repercussion (Table 1) on the extent of protein function, protein synthesis, or transport within the cell [31]. Kris De Boeck and Margarida Amaral’s classification of CFTR mutations suggests seven classes from class I to VII [4,32]. Other classification suggested that class VII should be added to class I under the name of subtype A (IA) based on the fact that there is no mRNA production in this type and clinical manifestations are as severe as in class I (subtype B) where no protein is produced [33]. Nevertheless, assigning mutations to a specific class is not as easy as it may seem; rather, it is very complicated. A single mutation could be assigned to many classes. The ΔF508 mutation, for instance, is a deletion mutation, which causes misfolding of the CFTR protein, followed by subsequent proteasome-mediated degradation, and results in improper chloride channel function. Additionally, this mutation also causes a deficiency in the translation of CFTR; thus, the ΔF508 mutation can at least be assigned to three classes, and may be assigned to classes II and III [34,35]. Another example is the R117H variant, which is classified as class IV, but could also belong to class III [31,36]. 

Mutations classified as Class I, II, and III are severe mutations, and are mostly linked to a classical or typical form of CF and cause hasty deterioration of respiratory function, extreme lung disease, and pancreatic insufficiency. On the other hand, mutations classified as Class IV to VI are mild mutations, and thus are associated with less severe phenotypes, pancreatic sufficiency, and are later characterized by bacterial colonization [37,38]. However, the loss of proper CFTR function as a chloride channel is common in all of the mutation classes. The classification of mutations into these classes provides a framework that aids in understanding the primary defect at a cellular level. Mutations belonging to the same class elucidate similar molecular and cellular effects and treatments. Variations in CFTR gene variants exhibit significant diversity among various ethnic populations. Research indicates that constructing a CFTR mutation spectrum specific to predominant mutations within distinct ethnic groups facilitates the diagnostic procedure [39,40].

### 5.1. Class I

In Class I mutations, encompassing those inducing incomplete or absent mRNA leading to nonfunctional protein synthesis, several mutation types are implicated, including frameshift, nonsense, and splicing mutations. Within Class IA, mutations provoke the production of unstable mRNA. Specifically, nonsense mutations instigate premature termination of mRNA synthesis, initiating the introduction of stop codons before the authentic stop codon. Subsequently, the mRNA is flagged as aberrant and swiftly subjected to degradation by the nonsense mRNA decay (NMD) surveillance system, ultimately resulting in its elimination. Within Class IB mutations, all alterations culminate in the synthesis of nonfunctional proteins. These proteins are promptly recognized and targeted for degradation by the proteasome machinery before their integration into the apical membrane [32].

### 5.2. Class II

Mutations that result in the formation of misfolded CFTR proteins are included in Class II. The misfolded protein is handled by the cell’s quality control system, leading to its ubiquitination in the endoplasmic reticulum. This flagged protein will be detected and degraded by proteasome, which prevents its transportation to apical membrane. This whole process is known as endoplasmic-reticulum-associated degradation or ERAD. The intricate regulation of abnormal CFTR biogenesis, protein folding, and degradation is governed by a multifaceted proteostatic network. Ongoing pharmacological investigations aim to further decipher the complexities of this network, with the ultimate goal of developing therapeutic interventions [32,41]. The class II allele, specifically the c.1521_1523delCTT mutation, commonly known as F508del, stands out as the predominant mutation, accounting for approximately 85% of clinical cystic fibrosis (CF) cases worldwide. However, the prevalence of class II mutations, aside from F508del, exhibits significant variability among populations. Particularly, Southern European populations and other regions demonstrate an increased frequency of such mutations [42]. Rare class II mutations, including c.3302T>A (M1101K), c.254G>A (G85E), c.1705T>G (Y569D), and c.3909C>G (N1303K), exhibit varying frequencies among CFTR2-registered CF patients, with prevalence rates of 0.2%, 0.7%, 0.03%, and 2.4%, respectively (source: cftr2.org). Previous studies have indicated that certain of these rare mutations, such as M1101K, G85E, and N1303K, display defects in CFTR processing akin to F508del-CFTR [43].

### 5.3. Class III

Impaired CFTR channel gating is a symptom of class III mutations. CFTR proteins with gating mutations show a dramatically reduced open probability, up to 100-fold lower than wild-type CFTR, despite the protein’s structural integrity and proper migration to the apical membrane. Abnormal ATP binding to the nucleotide-binding domains (NBD1 and NBD2) and the lack of ATP hydrolysis are the causes of this gating dysfunction. They take the form of missense mutations, which make it easier for a protein to be produced at normal quantities and successfully incorporated into the membrane. Nevertheless, they exhibit resistance to protein kinase A activation. Classical symptoms of CF disease result from a significant compromise in CFTR function. This tendency is highlighted by the most common class III mutation, G551D. The correction of the gating error with a single agent, known as a potentiator, is capable of partially restoring a large proportion of CFTR function, hence improving patient outcomes. However, they are resistant to activation by protein kinase A. A notable example is the p.Gly551Asp mutation, which eliminates ATP-dependent gating, resulting in a reduced open probability approximately 100-fold lower than that of the wild-type channel [2,32,44].

### 5.4. Class IV

These mutations, which are mostly found in membrane-spanning regions critical for channel pore formation, are predominantly mis-sense mutations. They produce a protein that is successfully incorporated into the membrane. The mutant CFTR maintains cAMP-dependent Cl^−^ channel activity, but has lower channel conductance, resulting in decreased chloride and bicarbonate conductance. Because some chloride or bicarbonate conductance is intact, people with class IV mutations frequently have milder CF illness symptoms. The most prevalent mutation in this category, R117H, introduces a gating (class III) deficiency, which is substantially alleviated by potentiator therapy with ivacaftor [36]. Furthermore, disease severity varies greatly among individuals with the F508del/R117H genotype due to changes in a polymorphic sequence within intron 9, which contains five, seven, or nine thymidine nucleotides (poly-T repeats). This variability increases the risk of exon-10 (formerly known as exon 9) being skipped. Notably, shorter poly-T repeats are associated with more severe CF illness symptoms. The case of the R117H mutation illustrates the challenges associated with using a CFTR mutation classification system to predict disease severity and treatment response [36]. The p.Arg117His mutation is the one in class IV that has been looked into the most. This mutant exhibits proper processing and generates cyclic AMP-regulated apical Cl^−^ currents, although patch-clamp research shows a reduction in channel-open probability and conductance [32,45].

### 5.5. Class V

Class V mutations lead to a notable decrease in functional CFTR at the apical membrane due to promoter mutations, alternate splicing defects, or missense mutations resulting in abnormal mRNA transcripts. The clinical manifestations within this class may exhibit variability among patients and within different organs of the same patients, but are generally mild [2]. The majority of class V mutations reduce the overall quantity of CFTR protein by influencing pre-mRNA splicing. These splice site alterations might result in the total or partial exclusion of an exon. In circumstances of partial exclusion, normal mRNA production continues, but at a lower level. When the missed exon aligns in phase, an incomplete nonfunctional channel is formed. However, when the skipped exon is out of phase, the emergence of a premature termination codon (PTC) causes fast destruction of the transcript via the NMD pathway [2,32]. Splicing mutations typically occur in introns at splice sites, disrupting critical splicing signals required for proper exon recognition (acceptor site, donor site, branch point, or polypyrimidine tract). Nonetheless, a rising proportion of mutations are found within exons. These exonic nucleotide changes can cause exon skipping by either disrupting Exonic Splicing Enhancer (ESE) motifs or creating Exon-Splicing Silencer (ESS) motifs [32,46].

### 5.6. Class VI

Class VI mutations produce a functional CFTR protein, which migrates to the plasma membrane but is unstable. Plasma membrane CFTR endocytosis or turnover is accelerated, thus reducing CFTR density and function. Class VI mutations were first described in 1999. In the original investigation, these mutations were detected in multiple heterozygous patients with F508del in one allele and a C-terminal mutation induced by either frameshift mutation (e.g., 4326delTC, 4279insA, and 4271delC) or premature stop codons (e.g., S1455X, Q1412X, and L1399X) in the other allele. The number of amino acids deleted ranges from 26 (S1455X) to 98 (4279insA), although only patients with more than 70 residues deleted (i.e., more deletion than Q1412X) show severe symptoms [47].

**Table 1:** CFTR mutation classification.

Classes	Class I		Class II	Class III	Class IV	Class V	Class VI
	IA	IB					
**CFTR Defect**	No mRNA synthesis	No Synthesis (Blocked Protein Production)	Misprocessing/Misfolding/Defective Trafficking	Regulation/Gating	Reduced Channel Conductance	Reduced Synthesis	Reduced Stability
**Mutation**	Frameshift, splicing, nonsense mutation	Canonical splices (mis-splice), frameshift, premature stop codon, nonsense (null)	Amino acid deletion, missense.	Missense, Amino acid change.	Missense, Amino acid change.	Missense, mis-splice (other splicing defects)	Missense, Amino acid change, nonsense, frameshift
**Mechansim**	Premature termination/ stop codon ↓ Unstable mRNA ↓ Degraded by Nonsense mRNA decay (NMD) surveillance system	Premature termination/stop codon in mRNA ↓ Formation of small quantities or severely defective or truncated CFTR protein ↓ Rapidly degraded by the ER ↓ Near complete absence of functional protein in the apical cell membrane	Synthesis of a misprocessed/immature/misfolded protein that is only partially glycosylated ↓ Not released from the ER and when prematurely released, rapidly degraded by the ubiquitin–proteasomal pathway ↓ The absence of mature CFTR and a small quantity of immature protein is transported to the apical membrane of the epithelial cell which does not function properly as a chloride channel	Normal, correctly folded CFTR is synthesized and trafficked to the apical membrane ↓ Activation/regulation by cAMP and ATP are disturbed ↓ Opening time of the channel is significantly reduced	CFTR is properly synthesized and transported, responds to cAMP and ATP ↓ Channel conductance is greatly reduced.	Slightly improper splicing ↓ Defective/inefficient trafficking ↓ Decreased synthesis of fully active CFTR	Properly synthesized CFTR ↓ Increased endocytosis or abnormal recycling ↓ Reduced Stability/Destabilized Protein
**Examples**	Dele2,3 (21 kb), 1717-1G→A	3950delT, G542X, R553X W1282X,	Arg560Thr, Asn1303Lys, Ile507del, Phe508del	G551D, Gly178Arg, Gly551Ser, Ser549Asn	R117H, R334W, G314E,R347P, D1152H, R117C	3849 + 10 kb C>T, 3272-26 A>G, 3849–10 kb CT, A455E	1811 + 1.6 kb A>G, 4326delTC, Gln1412X, 4279insA
**References**	[32,33]	[19,29,31,38]	[19,38,48]	[19,38,48]	[19,29,48]	[19,29,38,48]	[19,29,31]

## 6. CFTR Modulators

CFTR modulators aim to correct the basic defect in the CFTR channel based on the type and nature of mutation. Modulators include amplifiers, correctors, potentiators, stabilizers, and read-through agents [8,49]. Potentiators improve channel gating, correctors improve CFTR misfolding and trafficking, while read-through drugs treat mutations that form in frame premature stop codons [49,50]. These modulators can also be called “targeted” or “mutation-specific” approaches. However, mutation-specific therapies are not very efficient for a couple of reasons: (1) one mutation may be classified into more than a single class (have pleiotropic defects), (2) many mutations are yet to be characterized, and (3) although mutations belong to one class, they may respond differently to the same treatment [51].

### 6.1. Class I Defects

Drugs correcting Class I CFTR mutations are called ‘premature stop codon suppressors’ or ‘read-through agents’ [52]. Class I modulators commonly include aminoglycoside antibiotics such as gentamicin, as well as non-aminoglycosides such as ataluren (PTC124) [50].

The use of gentamicin as an effective bactericidal aminoglycoside was described around 15 years ago, and it was then that it was discovered that gentamicin is capable of surpassing premature stop codons and allowing for the synthesis of a completely functional CFTR protein [19,53]. Recently, the benefits of the nasal application of gentamicin have also been observed [8,54,55]. Similarly, Ataluren is a small molecule that allows for the read-through of stop codons and permits the transcription and synthesis of full-length CFTR despite nonsense mutations [56]. Oral administration of ataluren has shown beneficial impacts on children and adults, with at least one Class I CFTR mutation, by significantly correcting CFTR function as a chloride channel in the respiratory cells [53]. Ataluren efficacy was checked with 14-day, 28-day, and 3-month trials administrating Ataluren. The Ataluren dosing regimens have shown improvements in chloride transport in 67% of the patients. It is not FDA-approved, but flavored sachets were used for study purposes [57]. In recent trial studies, two 48-week trial studies including 517 patients with at least one nonsense class I mutation compared ataluren to a placebo. Results showed no improvement in lung function and sweat chloride levels, and kidney damage was more common in people using ataluren [58].

### 6.2. Class II Defects

CFTR modulators aimed at Class II include chemical and molecular chaperones that can potentially improve CFTR protein folding and trafficking [50]. Treatments directed at Class II mutations are incredibly important, as they aim to correct the most abundant mutation ΔF508 falls in this class. Effective treatment of the ΔF508 mutation must enhance both CFTR channel gating and membrane stability [19]. Substances that alter and improve the ΔF508 trafficking to the membrane are referred to as “correctors”, whereas those that can increase the PKA-regulated open probability of the channel are known as “potentiators” [59,60]. For treating class II mutations, a combination of two types of modulators, correctors and potentiators, will be preferred. VX-809 (lumacaftor), ivacaftor, and tezacaftor showed significant results in monotherapy. Lumacftor is the first drug approved for treating PwCF that is homozygous for F508del mutation (Pecoraro, Serra, Pascale, and Franceschelli, 2023). VX-809 is an intriguingly essential corrector that has enhanced the maturation process in CFTR mutations in human bronchial epithelial cells in culture (Clancy et al., 2012). Another modulator similar to the VX-809, known as VX-661, is also under trial [50,61]. PTI (posenacaftor) rescues Phe508del-CFTR processing, PM trafficking, and channel function. This activity is enhanced when VX-661 and VX-809 is combined with PTI, but no such results were obtained when combined with VX-445. PTI and VX-445 are both correctors that act using same mechanism of action [62].

Insilco’s study showed that the I1234V mutation can be classified as a class II mutation after visualizing its misfolded CFTR protein structure l [63]. Modulator therapy for this class includes ETI (Elexacaftor/Tezacaftor/Ivacaftor) combined therapy. A recent study conducted on seven patients with I1234V mutations and no F508del mutation showed that sometimes Insilco predictions might be contradictory to actual results, as it predicted no improvement with combined ETI (Elexacaftor/Tezacaftor/Ivacaftor) therapy, but ETI therapy on patients showed significant improvement in channel activity [64]. Significant clinical improvements, reduced sweat chloride levels, and a decline in routine serum inflammatory markers were the outcomes when the patients who were heterozygous for F508del treated with ETI combined therapy. Moreover, there was a significant decrease in the amounts of TNF, IL-6, IL-18, and IL-1β in both stimulated peripheral blood mononuclear cells (PBMCs) and CF serum [65]. ETI treatment for one year has showed significant improvement in BMI, serum biochemical parameters of liver function, and LDL cholesterol level in patients with homozygous f508del. The results showed somewhat equal level of improvement, as shown in 3 year therapy with LI [66]. 

### 6.3. Class III Defects

Modulators known as CFTR channel activators or potentiators typically help with the treatment of Class III mutations. Channel Gating Potentiators such as VX-770 (ivacaftor) have been used to treat patients with at least one G551D mutation. This treatment regimen produced the activation of chloride transport via CFTR in epithelial cells bearing the G551D mutation [67,68]. Ivacaftor is the first drug in the market aimed at restoring the defective protein function, and has also been approved by U.S. regulators [69]. It is designed to improve the defective function of CFTR by enhancing channel gating [70]. Although the mechanism of ivacaftor is not completely elucidated or understood yet, it is assumed that the drug can restore the cAMP-dependent activity of surface CFTR mutant forms while allowing for minimal phosphorylation of the R domain [69]. Another naturally occurring flavonoid, genistein, has also been proven useful in therapies aimed at Class III and IV defects [71].

### 6.4. Class IV Defects

Class IV defects require compensation for the reduced CFTR conductance, which can be reached by increasing the overall amount of CFTR and channel activation; hence, both correctors and potentiators may be used [50]. Initially, it was assumed that Genistein works by inhibiting regulatory enzymes, particularly tyrosine kinase. However, not too long ago, it was proposed that genistein interacts directly with CFTR at an NBD, resulting in a higher chance of the chloride channel being in an open conformation [72]. Other therapeutic agents aimed at this class include benzimidazolone, Phloxine B, and GPact-11a, all of which stimulate CFTR channels or chloride currents [71]. R334W is a variant that causes a mild CF phenotype with a properly processed protein, but has reduced conductance. The efficacy of the PDE4 inhibitors Roflumilast and ivacaftor has been assessed. Both of these showed significant results in restoring R334W-CFTR channel activity. Combined therapy with these has further increased the recovery of channel activity [73].

### 6.5. Class V and VI Defects

Class V and VI defects affect mRNA splicing and produce unstable CFTR. Recent therapeutic advances have suggested using antisense oligonucleotides (AONs) for the specific correction of mis-splicing. Correctors aimed at these classes may include activators of Rac1 signaling, 4-PBA, milrinone, and genistein, all of which are agents that increase mRNA and maximize activation of the normal CFTR. A case report on ETI therapy of two patients with F508del/3849 + 10 kb C>T mutations has shown good results. Two female in their mid-thirties were treated with triple combination therapy, and their sweat chloride test value decreased, FEV1 value increased, and PEI was delayed [74]. G1244E mutant CFTR protein shows gating defects, and thus can be classified as a Class VI mutation. A patient with G1244E mutations showed poor results. Lumacaftor was mainly acting as a potentiator in heterologous models, but elexacftor increased the expression of mature CFTR in naïve cells. Overall, elexacftor in combined therapy could be a treatment option [75].

## 7. Monotherapy to Triple Combination Therapy (ETI)

The STRIVE and ENVISION phase 3 clinical trials showed ivacaftor—the first CFTR potentiator—markedly improved lung function, nutritional status, and patient-reported respiratory symptoms in persons with CF having the G551D-CFTR mutation (Table 2). Similar advantages were observed in younger children in the KIWI study. Since then, ivacaftor has been authorized for a number of CFTR gating and residual function alterations, demonstrating its therapeutic efficacy for a range of age groups and mutations [76]. LUMA + IVA in PwCF ≥ 12 years homozygous for F508del-CFTR was investigated in two 24-week phase 3 trials, TRAFFIC and TRANSPORT. The results showed modest improvements in ppFEV1, BMI, CFQ-R scores, and reduced pulmonary exacerbations. On the other hand, the therapy group saw a greater rate of discontinuation because of its side-effects, such as increased liver function, hemoptysis, and creatine kinase. Subsequent research in children aged 6 to 11 revealed notable gains in lung function, but not in BMI or CFQ-R ratings; several unfavorable side-effects forced the trial’s termination. Although the effectiveness of the combination therapy is recognized, its usage is restricted due to concerns about drug interactions and safety [62,76,77,78,79].

A phase 2 trial evaluated TEZA-IVA in PwCF homozygous for F508del-CFTR and compound heterozygous for F508del- and G551D-CFTR. The results demonstrated that TEZA-IVA was safer and improved lung function more than LUMA-IVA, and it was also well tolerated with low discontinuation rates. Compared to IVA monotherapy in compound heterozygous patients, TEZA-IVA significantly improved SCC and lung function in PwCF homozygous for F508del-CFTR. These results were validated with the EVOLVE phase 3 trial, which showed a significant increase in ppFEV1 and a decrease in pulmonary exacerbations in PwCF ≥ 12 years old who were homozygous for F508del-CFTR. The EXPAND phase 3 trial demonstrated significant improvements in ppFEV1 and better outcomes than IVA monotherapy without serious respiratory adverse effects, extending these advantages to those who were heterozygous for F508del-CFTR with a residual function mutation. Label extensions for other CFTR mutations have been granted by the FDA based on in vitro studies, much as IVA monotherapy [80,81,82].

The TEZA-IVA combination showed only moderate efficacy in PwCF homozygous for F508del-CFTR or heterozygous with a second CFTR residual function mutation, despite advancements in CF treatments. PwCF heterozygous for F508del-CFTR with a limited function mutation on the second allele showed considerably fewer clinical advantages. Through methods different from LUMA and TEZA, two novel correctors, VX-659 and elexacaftor, showed that they could rescue F508del-CFTR folding and trafficking to the plasma membrane. Subsequently, these correctors were assessed in triple combinations with TEZA-IVA in phase 2 and 3 trials [83,84]. ETI has been so far the best modulator therapy in rescuing F508del CFTR protein and restoring its function. Despite its promising results in cystic fibrosis patients, some studies shows ETI might show adverse effects in few cases. Studies are being conducted to fully understand ETI effects on cystic fibrosis-related problems such as inflammation, glucose level, microbial infection, etc. Some of the recent studies are listed below [85].

**Table 2:** Effect of ETI therapy on cystic fibrosis-related problems.

Study	Study Population	Problem Targeted	Conclusions
[86]	34 adults with CF and at least one F508del. 3–12 months. 23 patient completed the study.	Cystic fibrosis related glycemia (CFRG).	Improvement in CGM derived measure of hyperglycemia and glycemic variability. No significant effect on hypoglycemia.
[87]	30 PW homozygous for F508del or at least one F508del.	Airway and systemic inflammation.	Reduced circulating concentration of IL-6, sTNFR1 and CRP shows effect on systemic inflammation. Sputum production diminished in two-thirds of the population after 1 year of therapy.
[65]	19 patient heterozygous for F508del and modulator naïve.	Systemic and immune cell-derived inflammatory cytokines level response to ETI.	Downregulation of routine serum inflammatory markers, reduced sweat chloride concentrations, as well as a significant reduction in both CF serum and stimulated PBMCs IL-18, IL-1β, TNF, and IL-6 levels.
[88]	21 PwCF.	Changes in sputum proteome.	Mean FEV1 value improved by 13.7% after ETI therapy. Sputum proteome shifted towards intermediate state different from both CF and normal control.
[89]	127 PwCF.	Iron status in PwCf.	Significant improvement in mean iron level (increased by 20.24 μg/dL), and ferratin level increased by 31.4%.
[90]	25 PwCF, 13 homozygous for F508del, 12 heterzygous for F508del.	Nasal nitric oxide level.	Nasal NO level showed significant increase after several month even reached normal level.
[91]	29 PwCF, 15 homozygous for F508del, 14 heterzygous for F508del.	Nutritional status and digestive function.	Data showed improvements in nutritional parameters, improved defecation, lower pancreatic substitution requirements, and improvements in exocrine pancreatic function (mutation specific).

## Data Availability

All data produced or generated during study has been given in the manuscript.

## References

[1] Rohlfs E.M., Zhou Z., Heim R.A., Nagan N., Rosenblum L.S., Flynn K., Scholl T., Akmaev V.R., Sirko-Osadsa D.A., Allitto B.A. (2011). Cystic fibrosis carrier testing in an ethnically diverse US population. Clin. Chem..

[2] Fanen P., Wohlhuter-Haddad A., Hinzpeter A. (2014). Genetics of cystic fibrosis: CFTR mutation classifications toward genotype-based CF therapies. Int. J. Biochem. Cell Biol..

[3] Ong T., Ramsey B.W. (2023). Cystic fibrosis: A review. JAMA.

[4] De Boeck K., Amaral M.D. (2016). Progress in therapies for cystic fibrosis. Lancet Respir. Med..

[5] Sheema, Bashir K., Fiaz S., Khan A.W., Haqqani S., Bibi A., Nawaz K., Khan M.A., Ullah A. (2024). Molecular identification of HCV genotypes among injecting drug users having HCV and HIV co-infection. Bull. Biol. Allied Sci. Res..

[6] Ullah I., Ullah A., Rehman S., Ullah S., Ullah H., Haqqni S., Amir M., Gul F., Bashir K. (2023). Prevalence and risk factors of helicobacter pylori infection among individuals with tobacco consumption habits in district Peshawar: A cross-sectional study. Bull. Biol. Allied Sci. Res..

[7] Green D.M., Lahiri T., Raraigh K.S., Ruiz F., Spano J., Antos N., Bonitz L., Christon L., Gregoire-Bottex M., Hale J.E. (2024). Cystic Fibrosis Foundation Evidence-Based Guideline for the Management of CRMS/CFSPID. Pediatrics.

[8] Clancy J.P., Cotton C.U., Donaldson S.H., Solomon G.M., VanDevanter D.R., Boyle M.P., Gentzsch M., Nick J.A., Illek B., Wallenburg J.C. (2019). CFTR modulator theratyping: Current status, gaps and future directions. J. Cyst. Fibros..

[9] Hwang T.-C., Yeh J.-T., Zhang J., Yu Y.-C., Yeh H.-I., Destefano S. (2018). Structural mechanisms of CFTR function and dysfunction. J. Gen. Physiol..

[10] Csanády L., Vergani P., Gadsby D.C. (2019). Structure, gating, and regulation of the CFTR anion channel. Physiol. Rev..

[11] Ramananda Y., Naren A.P., Arora K. (2024). Functional Consequences of CFTR Interactions in Cystic Fibrosis. Int. J. Mol. Sci..

[12] Levring J., Chen J. (2024). Structural identification of a selectivity filter in CFTR. Proc. Natl. Acad. Sci. USA.

[13] Robert R., Norez C., Becq F. (2005). Disruption of CFTR chloride channel alters mechanical properties and cAMP-dependent Cl^−^ transport of mouse aortic smooth muscle cells. J. Physiol..

[14] Decherf G., Bouyer G., Egée S., Thomas S.L. (2007). Chloride channels in normal and cystic fibrosis human erythrocyte membrane. Blood Cells Mol. Dis..

[15] Lamhonwah A.M., Bear C.E., Huan L.J., Chiaw P.K., Ackerley C.A., Tein I. (2010). Cystic fibrosis transmembrane conductance regulator in human muscle: Dysfunction causes abnormal metabolic recovery in exercise. Ann. Neurol..

[16] Linsdell P. (2006). Mechanism of chloride permeation in the cystic fibrosis transmembrane conductance regulator chloride channel. Exp. Physiol..

[17] Nawaz K., Khan S., Bibi A. (2024). Insights into scabies prevalence and risk factors. Bull. Biol. Allied Sci. Res..

[18] Ullah A., Bibi A., Ullah I., Kayani R.E.Z., Asim M., Munawar N., Amjad M., Siraj M., Gohar M., Khan M.A. (2024). An overview of hepatitis C virus and liver cirrhosis in Pakistan. Bull. Biol. Allied Sci. Res..

[19] Lubamba B., Dhooghe B., Noel S., Leal T. (2012). Cystic fibrosis: Insight into CFTR pathophysiology and pharmacotherapy. Clin. Biochem..

[20] Ishiguro H., Steward M.C., Naruse S., Ko S.B., Goto H., Case R.M., Kondo T., Yamamoto A. (2009). CFTR functions as a bicarbonate channel in pancreatic duct cells. J. Gen. Physiol..

[21] Tang L., Fatehi M., Linsdell P. (2009). Mechanism of direct bicarbonate transport by the CFTR anion channel. J. Cyst. Fibros..

[22] Garcia M.A.S., Yang N., Quinton P.M. (2009). Normal mouse intestinal mucus release requires cystic fibrosis transmembrane regulator–dependent bicarbonate secretion. J. Clin. Investig..

[23] O’Riordan T.G., Donn K.H., Hodsman P., Ansede J.H., Newcomb T., Lewis S.A., Flitter W.D., White V.S., Johnson M.R., Montgomery A.B. (2014). Acute hyperkalemia associated with inhalation of a potent ENaC antagonist: Phase 1 trial of GS-9411. J. Aerosol Med. Pulm. Drug Deliv..

[24] Borowitz D. (2015). CFTR, bicarbonate, and the pathophysiology of cystic fibrosis. Pediatr. Pulmonol..

[25] Bernard A., Hermans C., Dom G., Terryn S., Leal T., Lebecque P., Cassiman J.-J., Scholte B.J., de Jonge H.R., Courtoy P.J. (2007). Cystic fibrosis is associated with a defect in apical receptor–mediated endocytosis in mouse and human kidney. J. Am. Soc. Nephrol..

[26] Raggi C., Fujiwara K., Leal T., Jouret F., Devuyst O., Terryn S. (2011). Decreased renal accumulation of aminoglycoside reflects defective receptor-mediated endocytosis in cystic fibrosis and Dent’s disease. Pflügers Arch. Eur. J. Physiol..

[27] Moran O. (2017). The gating of the CFTR channel. Cell. Mol. Life Sci..

[28] Wang W., El Hiani Y., Linsdell P. (2011). Alignment of transmembrane regions in the cystic fibrosis transmembrane conductance regulator chloride channel pore. J. Gen. Physiol..

[29] Bergeron C., Cantin A.M. (2019). Cystic fibrosis: Pathophysiology of lung disease. Seminars in Respiratory and Critical Care Medicine.

[30] Wang X.R., Li C. (2014). Decoding F508del misfolding in cystic fibrosis. Biomolecules.

[31] Espel J.C., Palac H.L., Bharat A., Cullina J., Prickett M., Sala M., McColley S.A., Jain M. (2018). The relationship between sweat chloride levels and mortality in cystic fibrosis varies by individual genotype. J. Cyst. Fibros..

[32] Bergeron C., Cantin A.M. (2021). New Therapies to Correct the Cystic Fibrosis Basic Defect. Int. J. Mol. Sci..

[33] Marson F.A.L., Bertuzzo C.S., Ribeiro J.D. (2016). Classification of CFTR mutation classes. Lancet Respir. Med..

[34] Lazrak A., Fu L., Bali V., Bartoszewski R., Rab A., Havasi V., Keiles S., Kappes J., Kumar R., Lefkowitz E. (2013). The silent codon change I507-ATC→ ATT contributes to the severity of the ΔF508 CFTR channel dysfunction. FASEB J..

[35] Cutting G.R., Engelhardt J., Zeitlin P.L. (2019). Genetics and pathophysiology of cystic fibrosis. Kendig’s Disorders of the Respiratory Tract in Children.

[36] Yu Y.C., Sohma Y., Hwang T.C. (2016). On the mechanism of gating defects caused by the R117H mutation in cystic fibrosis transmembrane conductance regulator. J. Physiol..

[37] Scotet V., L’hostis C., Férec C. (2020). The changing epidemiology of cystic fibrosis: Incidence, survival and impact of the CFTR gene discovery. Genes.

[38] Castellani C., Assael B.M. (2017). Cystic fibrosis: A clinical view. Cell. Mol. Life Sci..

[39] Schrijver I., Pique L., Graham S., Pearl M., Cherry A., Kharrazi M. (2016). The Spectrum of CFTR Variants in Nonwhite Cystic Fibrosis Patients: Implications for Molecular Diagnostic Testing. J. Mol. Diagn. JMD.

[40] Sugarman E.A., Rohlfs E.M., Silverman L.M., Allitto B.A. (2004). CFTR mutation distribution among U.S. Hispanic and African American individuals: Evaluation in cystic fibrosis patient and carrier screening populations. Genet. Med. Off. J. Am. Coll. Med. Genet..

[41] Estabrooks S., Brodsky J.L. (2020). Regulation of CFTR biogenesis by the proteostatic network and pharmacological modulators. Int. J. Mol. Sci..

[42] Bobadilla J.L., Macek M., Fine J.P., Farrell P.M. (2002). Cystic fibrosis: A worldwide analysis of CFTR mutations—Correlation with incidence data and application to screening. Hum. Mutat..

[43] Laselva O., Bartlett C., Gunawardena T.N., Ouyang H., Eckford P.D., Moraes T.J., Bear C.E., Gonska T. (2021). Rescue of multiple class II CFTR mutations by elexacaftor+ tezacaftor+ ivacaftor mediated in part by the dual activities of elexacaftor as both corrector and potentiator. Eur. Respir. J..

[44] Accurso F.J., Rowe S.M., Clancy J., Boyle M.P., Dunitz J.M., Durie P.R., Sagel S.D., Hornick D.B., Konstan M.W., Donaldson S.H. (2010). Effect of VX-770 in persons with cystic fibrosis and the G551D-CFTR mutation. N. Engl. J. Med..

[45] Keenan K., Dupuis A., Griffin K., Castellani C., Tullis E., Gonska T. (2019). Phenotypic spectrum of patients with cystic fibrosis and cystic fibrosis-related disease carrying p. Arg117His. J. Cyst. Fibros..

[46] Bareil C., Bergougnoux A. (2020). CFTR gene variants, epidemiology and molecular pathology. Arch. Pédiatr..

[47] Yeh J.T., Yu Y.C., Hwang T.C. (2019). Structural mechanisms for defective CFTR gating caused by the Q1412X mutation, a severe Class VI pathogenic mutation in cystic fibrosis. J. Physiol..

[48] Boyle M.P., De Boeck K. (2013). A new era in the treatment of cystic fibrosis: Correction of the underlying CFTR defect. Lancet Respir. Med..

[49] Lopes-Pacheco M. (2020). CFTR Modulators: The Changing Face of Cystic Fibrosis in the Era of Precision Medicine. Front. Pharmacol..

[50] Bell S.C., De Boeck K., Amaral M.D. (2015). New pharmacological approaches for cystic fibrosis: Promises, progress, pitfalls. Pharmacol. Ther..

[51] Lukacs G.L., Verkman A. (2012). CFTR: Folding, misfolding and correcting the ΔF508 conformational defect. Trends Mol. Med..

[52] Derichs N. (2013). Targeting a genetic defect: Cystic fibrosis transmembrane conductance regulator modulators in cystic fibrosis. Eur. Respir. Rev..

[53] Kerem E., Hirawat S., Armoni S., Yaakov Y., Shoseyov D., Cohen M., Nissim-Rafinia M., Blau H., Rivlin J., Aviram M. (2008). Effectiveness of PTC124 treatment of cystic fibrosis caused by nonsense mutations: A prospective phase II trial. Lancet.

[54] Sermet-Gaudelus I., Renouil M., Fajac A., Bidou L., Parbaille B., Pierrot S., Davy N., Bismuth E., Reinert P., Lenoir G. (2007). In vitro prediction of stop-codon suppression by intravenous gentamicin in patients with cystic fibrosis: A pilot study. BMC Med..

[55] Sermet-Gaudelus I., Boeck K.D., Casimir G.J., Vermeulen F., Leal T., Mogenet A., Roussel D., Fritsch J., Hanssens L., Hirawat S. (2010). Ataluren (PTC124) induces cystic fibrosis transmembrane conductance regulator protein expression and activity in children with nonsense mutation cystic fibrosis. Am. J. Respir. Crit. Care Med..

[56] Welch E.M., Barton E.R., Zhuo J., Tomizawa Y., Friesen W.J., Trifillis P., Paushkin S., Patel M., Trotta C.R., Hwang S. (2007). PTC124 targets genetic disorders caused by nonsense mutations. Nature.

[57] Pettit R.S., Fellner C. (2014). CFTR Modulators for the Treatment of Cystic Fibrosis. P&T Peer-Rev. J. Formul. Manag..

[58] Aslam A.A., Sinha I.P., Southern K.W. (2023). Ataluren and similar compounds (specific therapies for premature termination codon class I mutations) for cystic fibrosis. Cochrane Database Syst. Rev..

[59] Dormer R.L., Harris C.M., Clark Z., Pereira M.M.C., Doull I.J.M., Norez C., Becq F., McPherson M.A. (2005). Sildenafil (Viagra) corrects ΔF508-CFTR location in nasal epithelial cells from patients with cystic fibrosis. Thorax.

[60] Luciani A., Villella V.R., Esposito S., Brunetti-Pierri N., Medina D., Settembre C., Gavina M., Pulze L., Giardino I., Pettoello-Mantovani M. (2010). Defective CFTR induces aggresome formation and lung inflammation in cystic fibrosis through ROS-mediated autophagy inhibition. Nat. Cell Biol..

[61] Murphy M.P., Caraher E. (2016). Current and emerging therapies for the treatment of cystic fibrosis or mitigation of its symptoms. Drugs R&D.

[62] Ferreira F.C., Amaral M.D., Bacalhau M., Lopes-Pacheco M. (2024). PTI-801 (posenacaftor) shares a common mechanism with VX-445 (elexacaftor) to rescue p. Phe508del-CFTR. Eur. J. Pharmacol..

[63] Aluma B.E.B., Sarouk I., Senderowitz H., Cohen-Cymberknoh M., Khazanov N., Dagan A., Bezalel Y., Ashkenazi M., Keler S., Efrati O. (2020). Phenotypic and molecular characteristics of CF patients carrying the I1234V mutation. Respir. Med..

[64] Aluma B.E.B., Reiter J., Efrati O., Bezalel Y., Keler S., Ashkenazi M., Dagan A., Buchnik Y., Sadras I., Cohen-Cymberknoh M. (2024). Clinical efficacy of CFTR modulator therapy in people with cystic fibrosis carrying the I1234V mutation. J. Cyst. Fibros..

[65] Jarosz-Griffiths H.H., Gillgrass L., Caley L.R., Spoletini G., Clifton I.J., Etherington C., Savic S., McDermott M.F., Peckham D. (2024). Anti-inflammatory effects of elexacaftor/tezacaftor/ivacaftor in adults with cystic fibrosis heterozygous for F508del. PLoS ONE.

[66] Castaldo A., Gelzo M., Iacotucci P., Longobardi A., Taccetti G., Terlizzi V., Carnovale V. (2024). One year of treatment with elexacaftor/tezacaftor/ivacaftor in patients with cystic fibrosis homozygous for the F508del mutation causes a significant increase in liver biochemical indexes. Front. Mol. Biosci..

[67] De Boeck K., Munck A., Walker S., Faro A., Hiatt P., Gilmartin G., Higgins M. (2014). Efficacy and safety of ivacaftor in patients with cystic fibrosis and a non-G551D gating mutation. J. Cyst. Fibros..

[68] Van Goor F., Hadida S., Grootenhuis P.D., Burton B., Cao D., Neuberger T., Turnbull A., Singh A., Joubran J., Hazlewood A. (2009). Rescue of CF airway epithelial cell function in vitro by a CFTR potentiator, VX-770. Proc. Natl. Acad. Sci. USA.

[69] Pyle L.C., Ehrhardt A., Mitchell L.H., Fan L., Ren A., Naren A.P., Li Y., Clancy J., Bolger G.B., Sorscher E.J. (2011). Regulatory domain phosphorylation to distinguish the mechanistic basis underlying acute CFTR modulators. Am. J. Physiol. Lung Cell. Mol. Physiol..

[70] Yu H., Burton B., Huang C.-J., Worley J., Cao D., Johnson J.P., Urrutia A., Joubran J., Seepersaud S., Sussky K. (2012). Ivacaftor potentiation of multiple CFTR channels with gating mutations. J. Cyst. Fibros..

[71] Cai Z., Taddei A., Sheppard D.N. (2006). Differential sensitivity of the cystic fibrosis (CF)-associated mutants G551D and G1349D to potentiators of the cystic fibrosis transmembrane conductance regulator (CFTR) Cl–channel. J. Biol. Chem..

[72] Esposito S., Villella V.R., Ferrari E., Monzani R., Tosco A., Rossin F., D’Eletto M., Castaldo A., Luciani A., Silano M. (2019). Genistein antagonizes gliadin-induced CFTR malfunction in models of celiac disease. Aging.

[73] Latorre R.V., Calicchia M., Bigliardi M., Conti J., Kleinfelder K., Melotti P., Sorio C. (2024). Functional rescue of CFTR in rectal organoids from patients carrying R334W variant by CFTR modulators and PDE4 inhibitor Roflumilast. Respir. Investig..

[74] Stastna N., Pokojova E. (2023). Case report of two adults with F508del/3849+ 10 kb C> T genotype regaining exocrine pancreatic function following treatment with elexacaftor/tezacaftor/ivacaftor. J. Cyst. Fibros..

[75] Tomati V., Costa S., Capurro V., Pesce E., Pastorino C., Lena M., Sondo E., Di Duca M., Cresta F., Cristadoro S. (2023). Rescue by elexacaftor-tezacaftor-ivacaftor of the G1244E cystic fibrosis mutation’s stability and gating defects are dependent on cell background. J. Cyst. Fibros..

[76] Bacalhau M., Ferreira F.C., Silva I.A., Buarque C.D., Amaral M.D., Lopes-Pacheco M. (2023). Additive potentiation of R334W-CFTR function by novel small molecules. J. Pers. Med..

[77] Hassan N., Amin F., Bashir K., Irshad M., Jamil S., Munawar N., Haqqani H., Shabir H., Khan M. (2023). Antiviral response of drugs used against hbv patients of Khyber Pakhtunkhwa, Pakistan. Bull. Biol. Allied Sci. Res..

[78] Gohar M., Rehman I., Ahmad J., Ahmad F., Bashir K., Ikram S., Hassan N., Khan M., Ullah A. (2023). Prevalence of hepatitis b virus and genotypes in the region of khyber pakhtunkhwa Pakistan. Bull. Biol. Allied Sci. Res..

[79] Awan S.J., Fatima Z., Kamran S., Khan A.S., Fatima T., Imran S., Shabbir M., Nadeem S.I. (2024). GUAR Gum in therapeutics: A succinct exploration. Bull. Biol. Allied Sci. Res..

[80] Costa E., Girotti S., Pauro F., Leufkens H.G., Cipolli M. (2022). The impact of FDA and EMA regulatory decision-making process on the access to CFTR modulators for the treatment of cystic fibrosis. Orphanet J. Rare Dis..

[81] Rowe S.M., Jones I., Dransfield M.T., Haque N., Gleason S., Hayes K.A., Kulmatycki K., Yates D.P., Danahay H., Gosling M. (2020). Efficacy and safety of the CFTR potentiator icenticaftor (QBW251) in COPD: Results from a phase 2 randomized trial. Int. J. Chronic Obstr. Pulm. Dis..

[82] Taylor-Cousar J.L., Mall M.A., Ramsey B.W., McKone E.F., Tullis E., Marigowda G., McKee C.M., Waltz D., Moskowitz S.M., Savage J. (2019). Clinical development of triple-combination CFTR modulators for cystic fibrosis patients with one or two F508del alleles. ERJ Open Res..

[83] Davies J.C., Van de Steen O., van Koningsbruggen-Rietschel S., Drevinek P., Derichs N., McKone E.F., Kanters D., Allamassey L., Namour F., de Kock H. (2019). GLPG1837, a CFTR potentiator, in p. Gly551Asp (G551D)-CF patients: An open-label, single-arm, phase 2a study (SAPHIRA1). J. Cyst. Fibros..

[84] Keating D., Marigowda G., Burr L., Daines C., Mall M.A., McKone E.F., Ramsey B.W., Rowe S.M., Sass L.A., Tullis E. (2018). VX-445–tezacaftor–ivacaftor in patients with cystic fibrosis and one or two Phe508del alleles. N. Engl. J. Med..

[85] Bacalhau M., Camargo M., Magalhães-Ghiotto G.A.V., Drumond S., Castelletti C.H.M., Lopes-Pacheco M. (2023). Elexacaftor-Tezacaftor-Ivacaftor: A Life-Changing Triple Combination of CFTR Modulator Drugs for Cystic Fibrosis. Pharmaceuticals.

[86] Scully K.J., Marchetti P., Sawicki G.S., Uluer A., Cernadas M., Cagnina R.E., Kennedy J.C., Putman M.S. (2022). The effect of elexacaftor/tezacaftor/ivacaftor (ETI) on glycemia in adults with cystic fibrosis. J. Cyst. Fibros..

[87] Casey M., Gabillard-Lefort C., McElvaney O.F., McElvaney O.J., Carroll T., Heeney R.C., Gunaratnam C., Reeves E.P., Murphy M.P., McElvaney N.G. (2023). Effect of elexacaftor/tezacaftor/ivacaftor on airway and systemic inflammation in cystic fibrosis. Thorax.

[88] Maher R.E., Barry P.J., Emmott E., Jones A.M., Lin L., McNamara P.S., Smith J.A., Lord R.W. (2024). Influence of highly effective modulator therapy on the sputum proteome in cystic fibrosis. J. Cyst. Fibros..

[89] James A., Li G., List R., Lonabaugh K., Smith A.D., Barros A., Somerville L., Albon D. (2024). Analysis of iron status after initiation of elexacaftor/tezacaftor/ivacaftor in people with cystic fibrosis. Pediatr. Pulmonol..

[90] Pioch C.O., Ziegahn N., Allomba C., Busack L.M., Schnorr A.N., Tosolini A., Fuhlrott B.R., Zagkla S., Othmer T., Syunyaeva Z. (2024). Elexacaftor/tezacaftor/ivacaftor improves nasal nitric oxide in patients with cystic fibrosis. J. Cyst. Fibros..

[91] Stastna N., Kunovsky L., Svoboda M., Pokojova E., Homola L., Mala M., Gracova Z., Jerabkova B., Skrickova J., Trna J. (2024). Improved nutritional outcomes and gastrointestinal symptoms in adult cystic fibrosis patients treated with elexacaftor/tezacaftor/ivacaftor. Dig. Dis..

